# Lessening the adverse effect of the semivariogram model selection on an interpolative survey using kriging technique

**DOI:** 10.1186/s40064-016-2142-4

**Published:** 2016-04-29

**Authors:** Zakari Arétouyap, Philippe Njandjock Nouck, Robert Nouayou, Franck Eithel Ghomsi Kemgang, Axel Dorian Piépi Toko, Jamal Asfahani

**Affiliations:** Postgraduate School of Science, Technology and Geosciences, University of Yaounde I, P.O. Box 812, Yaounde, Cameroon; Applied Geophysics Division, Head Atomic Energy Commission, P.O. Box 6091, Damascus, Syria

**Keywords:** Interpolation, Kriging, Predictive analysis, Spatial analysis, Structural analysis, Semivariogram

## Abstract

**Objective:**

Many parameters in environmental, scientific and human sciences investigations need to be interpolated. Geostatistics, with its structural analysis step, is widely used for this purpose. This precious step that evaluates data correlation and dependency is performed thanks to semivariogram. However, an incorrect choice of a semivariogram model can skew all the prediction results. The main objectives of this paper are (1) to simply illustrate the influence of the choice of an inappropriate semivariogram model and (2) to show how a best-fitted model can be selected. This may lessen the adverse effect of the semivariogram model selection on an interpolation survey using kriging technique.

**Methods:**

The influence of the semivariogram model selection is highlighted and illustrated by thematic maps drawn using four different models (Gaussian, magnetic, spherical and exponential). Then, a guideline to select the most suitable model, using mean error (ME), mean square error (MSE), root mean square error (RMSE), average standard error (ASE), and root mean square standardized error (RMSSE), is proposed.

**Results:**

The choice of a semivariogram model seriously influences the results of a kriging survey at both endpoints and amplitude of the range of the estimated values. However, the direction of variation of the interpolated values is independent of the semivariogram model: different semivariogram models (with the same characteristics) produce different thematic maps but, the areas of minimum and maximum values remain unchanged. Yet, the suitable model can be selected by means of ME, MSE, RMSE, ASE and RMSSE.

**Conclusion:**

The present article illustrates how the use of an inappropriate semivariogram model can seriously distort the results of an evaluation, assessment or prediction survey. To avoid such an inconveniency, a methodical approach based on the computation and analysis of ME, RMSE, ASE, RMSSE and MSE is proposed.

## Background

Geostatistics is used to address various natural and human problems with a spatial dimension. Actually, Geomatics is one of the most important specialties because numerous of phenomena and matters studied in Geosciences need to be mapped in terms of simple illustration (reprography or presentation) or in terms of assessment (prediction or forecasting), management and allocation of the world’s physical and/or human resources. In particular, assessing a variable is very delicate because it is a matter of interpolating that variable where no measurement has been conducted or, establishing a correlation between data of different natures. For this purpose, several softwares have been developed including ArcGIS and Golden Surfer, and are being widely used by thousands of scientists worldwide for various aims.

Arétouyap et al. (2014a, b, 2015) used geostatistics to analyze the spatial distribution of climate parameters in central Africa, the groundwater quality index in the Adamwa-Cameroon region and to characterize aquifers in the Pan-African context; Binita et al. (2015) to investigate temporal and spatial assessment of climate change vulnerability; Chaney and Rojas-Guyler (2015) to establish the geographic variability in adolescent drug use and to correlate factors of use; Keumseok et al. (2015) to build up spatial patterns of simulated obesity prevalence were compared with measures of low income and food accessibility; Mishra and Chaudhuri (2015) to characterize spatio-temporal trends in vegetation greenness in Uttarakhand Himalayas; Zunkel (2015) to establish a network of all 14 tornado sirens and examined the number of residents included and not included in that network, Teikeu Assatse et al. (2016) to assess water quality.

Most of mentioned modellings, geospatializations and interpolations are conducted thanks to ArcGis and Golden Surfer. The functioning of these softwares is based on interpolative techniques such as Minimum Curve, Inverse Distance, Spline functions, Trend Surface and Kriging (Sacks and Schiller 1988). Kriging is distinguished from all these techniques through its unbiased feature. It is so called BLUE (Best Linear Unbiased Estimator). Thus, it is by far the most used method to that purpose in all domains of environmental sciences worldwide (Diodato et al. 2013, Arétouyap et al. 2014a, b; Nshagali et al. 2015; Teikeu Assatse et al. 2016). The use of this method is growing with the development of new mining platforms across the New Industrialized Countries (Cameroon, Australia, South Africa, Mexico, Ethiopia, Brazil, Turkey, Philippines, etc.).

This method so efficient, effective and popular with geoscientists has a very important preliminary step upon which the reliability of interpolation and prediction depends: that is the structural analysis focused on the semivariogram. This step is so important that for many versions of Golden Surfer and ArcGIS, it is of the responsibility of the user to select the suitable model of semivariogram. For this reason, van Groenigen (2000) studied the influence of semivariogram parameters on optimal sampling schemes for mapping by kriging; Cressie (1993) advised the use of cross-validation to check the validity of a semivariogram model; Crujeiras et al. (2001) derived the goodness-of-fit tests with this aim and, Gorsich and Genton (2000) introduced the use of nonparametric derivative estimation. The main objectives of this paper are (1) to simply illustrate the influence of the choice of an inappropriate semivariogram model and (2) to show how a best-fitted model can be selected. This may lessen the adverse effect of the semivariogram model selection on an interpolation survey using kriging technique.

## Results

### Descriptive statistics

The database used is made of 50 values of aquifer resistivity ranged from 3 to 852 Ω m, with a mean of 228 Ω m and a standard deviation (SD) of 218 Ω m. Table 1 summarized the distribution of the data.

**Table 1:** Descriptive statistics of the database

Parameter	Number	Min (Ω m)	Max (Ω m)	Mean (Ω m)	Median (Ω m)	SD (Ω m)	Skew	Kurtosis
Resistivity (Ω m)	50	190	280	228	166	218	1.06	0.41

No need to plot histogram, neither QQ plot to check data normality. Indeed, above Table 1 shows that the median is greater than the half mean value. This indicates that more or less normal distribution of data.

### Cross-validation

**Fig. 1:**
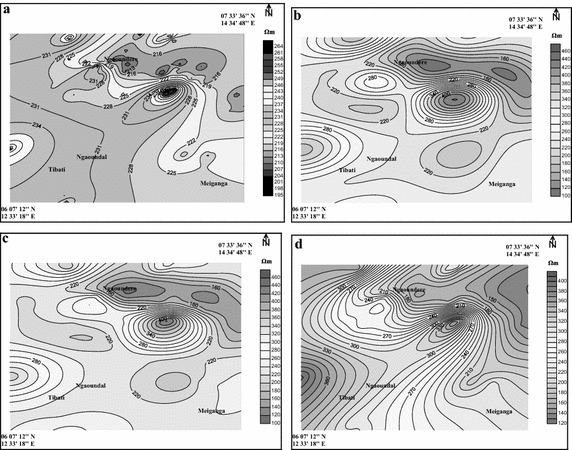
Thematic maps of estimation performed using different variogram models (**a** Gaussian model, **b** magnetic model, **c** spherical model, **d** exponential model). These maps are different each from the others

Using the Gaussian model, the estimated resistivity rages between 195 and 267 Ωm. The magnetic and spherical models produce values ranged from 100 to 480 Ωm while the exponential model provides a range of 120–420 Ωm. In general, each model produced a result different from each other. The difference may be in the endpoints of the range or its amplitude. These differences are summarized in Table 2 and illustrated in Fig. 1.

**Table 2:** Differences from analytical analysis between the four variogram models

	Gaussian	Exponential	Magnetic/spherical
Minimum	195	120	100
Maximum	267	420	480
Magnitude	72	300	380

Furthermore, to appreciate the functioning of cross-validation developed above, a well-known point value (200 Ω m-value obtained at location P-30) has been hidden, then estimated using different semivariogram models. Results summarized in Table 3 agree that Gaussian model provides the most accurate estimation.

**Table 3:** Illustration of the cross-validation test

Model	Experimental value, *R* (Ω m)	Estimated value $R^{*}$ (Ω m)	$\frac{{R^{*} - R}}{R}(\% )$	Comments
Gaussian	200	201	+0.5	Almost identical
Exponential	200	132	−34	Underestimated
Magnetic	200	143	−29	Underestimated
Spherical	200	98	−51	Very underestimated

## Discussion

In the particular case of this study, values interpolated using spherical and magnetic models ranged in the same interval (100–480 Ωm). But in general, each semivariogram model provides distinct result. However, despite their observed differences, all thematic maps have the same variation trend. The gradient values are constant: the minimum and maximum values are almost in the same regions respectively from one map to another.

These observations are in compliance with results published by many other authors (Webster and Oliver 2007; Chilès and Delfiner 2012). It is therefore evident that the quality and the reliability of an interpolation by kriging strongly depend on the structural analysis of field data, that is to say, the semivariogram model. Predictive performances of the fitted models are checked on the basis of cross-validation tests.

**Fig. 2:**
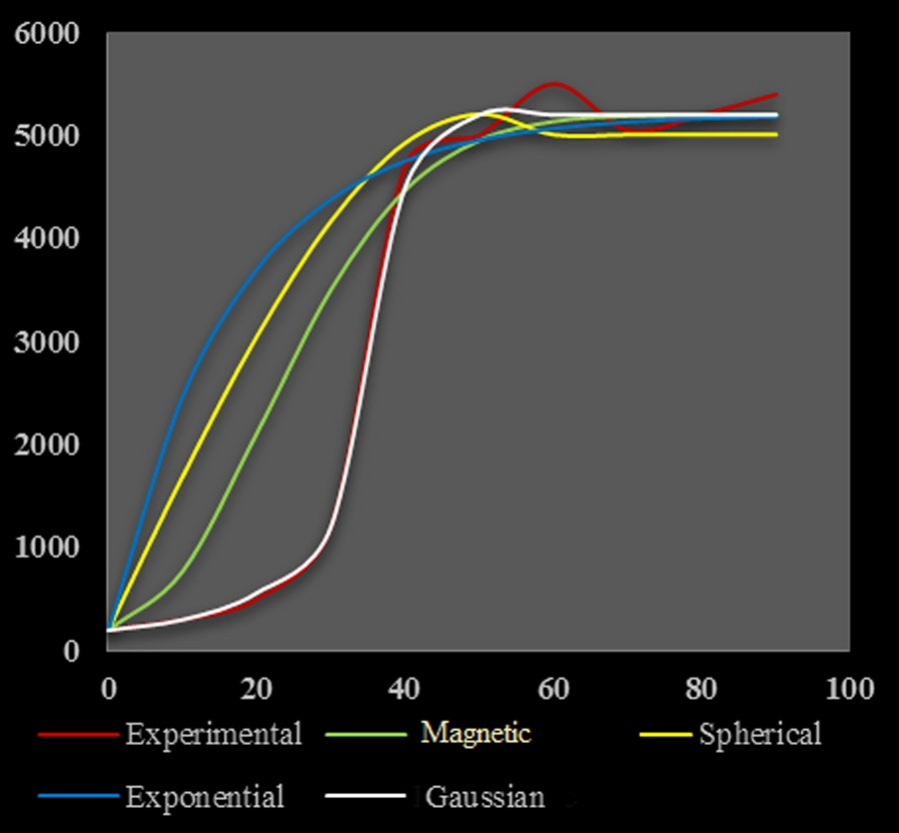
The four variogram models plotted together with the experimental one in order to highlight that the logarithmic model is best-fitted one

Table 4 shows that the Gaussian model is the best-fitted one. This agrees with Fig. 2 which illustrates that the same model (Gaussian) accommodates the most with the experimental semivariogram, although the serious concern the dataset is facing. Indeed, before you can use this statistical method based on the theory of regionalized variables, you must make a semivariogram model, which will determine the interpolation function. However, kriging is optimal when data are normally distributed and stationary i.e. mean and variance do not vary significantly in space (Isaaks and Srivastava 1989; Goovaerts 1997; Kitanidis 1997; Deutsch and Journel 1998; Webster and Oliver 2007).

**Table 4:** Analytical characteristics of semivariogram models used to detect the best-fitted one

	ME	RMSE	ASE	MSE	RMSSE
Gaussian	0.02	8.41	8.03	0.08	0.97
Magnetic	3.52	18.21	21.36	3.18	3.14
Spherical	5.24	20.07	23.21	7.01	3.20
Exponential	17.36	29.57	32.33	18.32	3.54

**Fig. 3:**
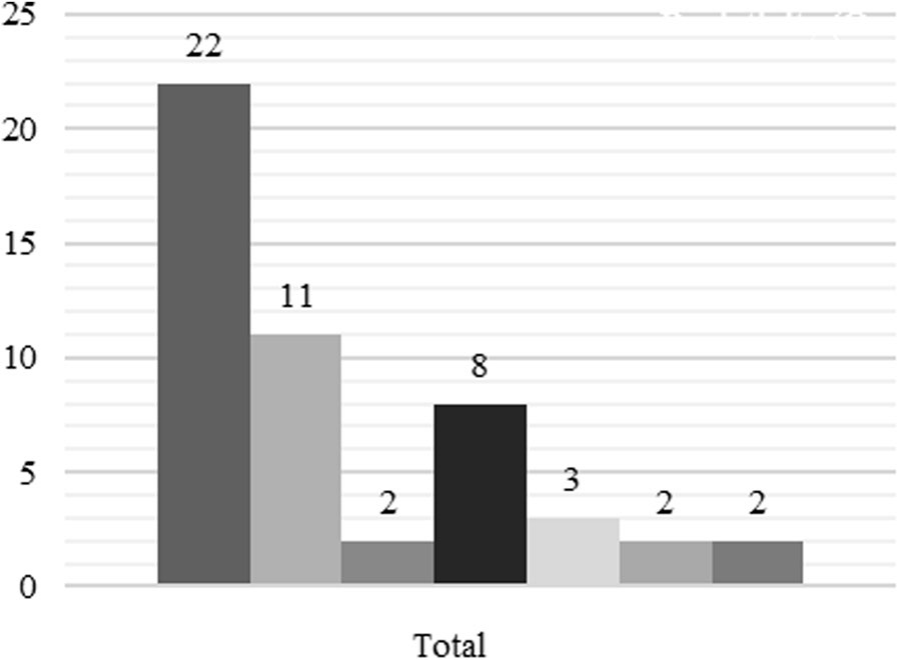
Histogram of resistivity data

In fact, as illustrated in Fig. 3, the data are not normally distributed as the histogram is no symmetrical. This condition can also be checked using quantile–quantile plot. To curb the impact of poor data distribution, we have introduced a lag tolerance of 10 km in order to get a reasonable number of pairs for computing statistics.

This study should have various applications and impacts on environmental and earth sciences. In fact, many environmental and earth deposits parameters are usually called to be predicted or estimated. However, one cannot carry out measurement continuously. The parameter to be estimated is measured discretely and then, to obtain the continuous information, kriging technique is used. Nowadays, this technique based on semivariogram is used by so many scientists in various fields as civil protection (Zamani and Mirabadi 2011), meteorology (Caridad and Jury 2013; Arétouyap et al. 2014a), geochemistry (Gorai and Kumar 2013; Méli’i et al. 2013; Arétouyap et al. 2014b; Nshagali et al. 2015; Arétouyap et al. 2015). If authors do not take into account the paramount impact of the semivariogram model in such investigations, the survey will be sketchy and results untruthful. This explains the importance of the present paper.

**Fig. 4:**
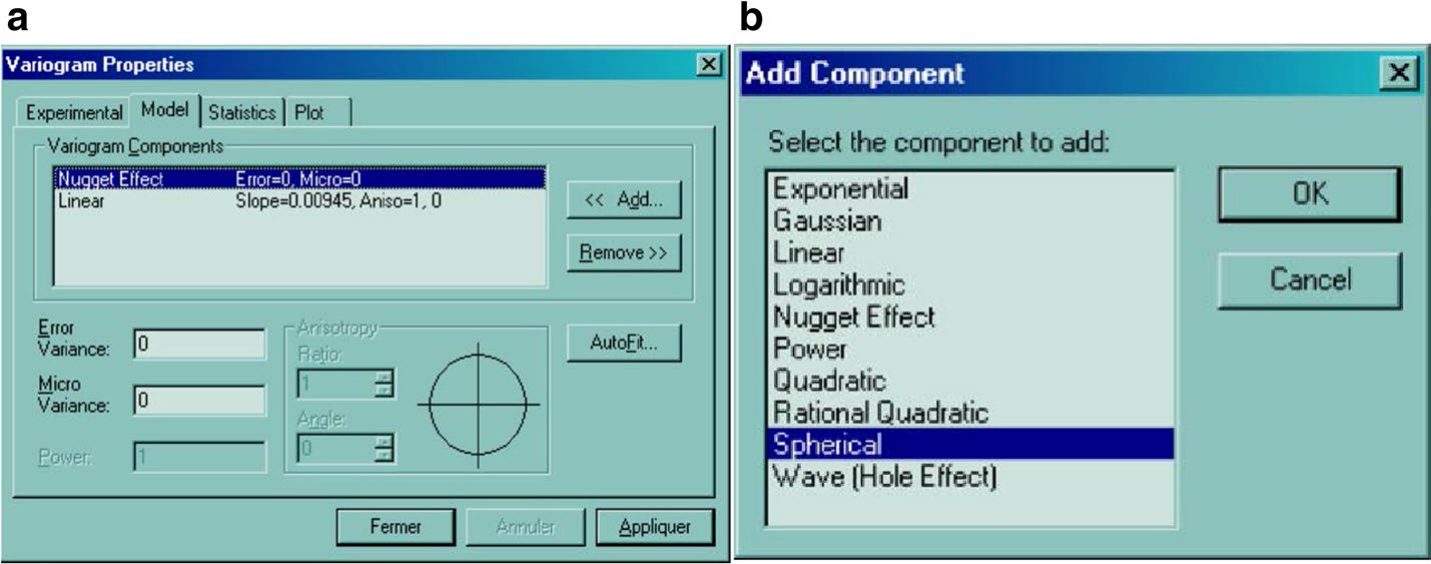
Selection of the suitable variogram model. A randon nugget or linear model is automatically proposed to the user (**a**), who should rationally select the appropriate one from the box (**b**)

Many other studies have been carried out in order to highlight the delicateness of modelling and assessment. Giuseppe and Petrarca (2013) bring up the effects of scale in spatial interaction models; Patuelli and Giuseppe (2013) published an editorial on the advances in the statistical modelling of spatial interaction data. But the present paper tackles the issue of the selection of the suitable semivariogram model. In fact, interpolation softwares automatically propose a random linear or nugget model to the user (Fig. 4a) and the user has to select and set up the best-fitted model (Fig. 4b).

When the random linear or nugget model is automatically displayed, the user should select and “add” a model that is suitable for his dataset, then fit it.

## Conclusion

**Fig. 5:**
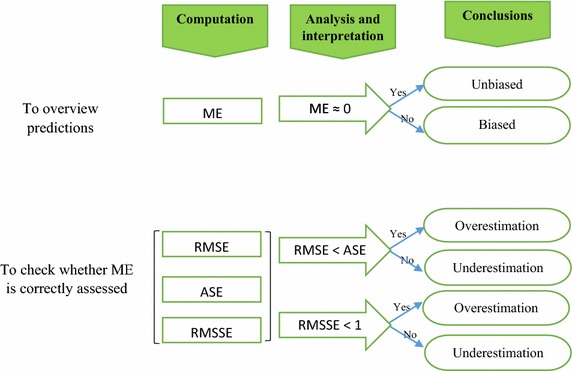
Illustration of the principle of a semivariogram model selection

The present paper highlighted and illustrated the adverse effect of the semivariogram model on a prediction or interpolation survey using kriging technique. An incorrect choice of a semivariogram model can skew the results of an evaluation, assessment or prediction survey. To avoid such an inconveniency, a methodical approach based on the computation and analysis of ME, RMSE, ASE, RMSSE and MSE is proposed and summarized by a chart (Fig. 5). This may be very useful for scientists and researchers who are called to solve various environmental, social and human problems. It is therefore necessary to well apply during the cross-validation test in order to select the best-fitted semivariogram model before predictive analysis.

## Methods

### Data used

In this experimental analysis, we used dataset of aquifer resistivity computed using the vertical electrical sounding conducted in the Pan-African context of Adamawa-Cameroon (Arétouyap et al. 2015). This field campaign was carried out in order to characterize local aquifers.

### Semivariogram and its influence on the kriging results

Currently, kriging is the best interpolation technique because it is unbiased. Nevertheless, it requires data to be correlated and dependent. This structural analysis is conducted by means of semivariogram.

The semivariogram is a mathematical function that is used to describe the spatial continuity of a phenomenon (Caridad and Jury 2013). In the present study, constant trend is observed at all locations. Data are said to be stationary. Hence, the theoretical formulation of the semivariogram *γ*(*h*), using the concept of variance (*Var*) applied to the difference between two observations *z*(*x*) and *z*(*x* + *h*) separated by a distance *h*, is expressed by Eq. 1.

1 $$\gamma (h) = \frac{1}{2}\text{var} \left[ {z(x) - z(x + h)} \right]$$

In practice, only the experimental semivariogram *γ*_*e*_(*r*) is calculated from observations using Eq. 2.

2 $$\gamma (h) = \frac{1}{2N(h)}\sum\limits_{i = 1}^{N(h)} {\left[ {z(x_{i} ) - z(x_{i} + h)} \right]^{2} }$$

where *γ*_*e*_(*h*) is the estimated value of the semivariogram for lag (*h*); N(h), the number of pairs of points separated by distance h; *z*(*x*_*i*_) and *z*(*x*_*i*_ + *h*) are values of *z* at positions *x*_*i*_ and *x*_*i*_ + *h*, respectively.

Ideally, a point of the experimental semivariogram is considered as representative if *N*(*h*) ≥ 30. At these point values, a suitable theoretical semivariogram model is adjusted. Nevertheless, theoretical models of semivariograms were adjusted in the present investigation despite the limited number of points (*N*(*h*) = 25). The main current eligible models are nugget effect, linear, gravimetric, cubic, pentaspherical, spherical, exponential, power, Gaussian, Cauchy and logarithmic semivariograms. A model is admissible if any variance calculated from the model is positive (Chilès and Delfiner 2012).

**Fig. 6:**
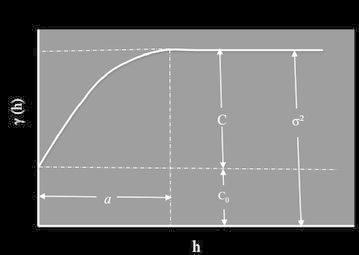
Experimental variogram

The description of a semivariogram model is based on the quantification of multiple parameters identified in Fig. 6. The range (length) *a* is the distance where the correlation between observations becomes zero. At this distance, the semivariogram reaches the sill (scale) *σ*^2^ which is the sum of the nugget variance *C*_0_ and the partial sill (variance) *C*. The nugget effect derives from various sources such as measurement errors, existence of a microstructure smaller than the size of the sample and/or the presence of a microstructure with a range less than the distance between the two closest observations. It may be impossible to quantify the contribution of each source.

To highlight the influence of the semivariogram model on the kriging results, four different semivariogram models (magnetic, Gaussian, exponential and spherical) with the same effect nugget (C_0_ = 200 Ω^2^ m^2^), the same sill (σ^2^ = 5200 Ω^2^ m^2^) and the same range (a = 50 m) were used to interpolate the data by kriging. These semivariogram models are expressed by Eqs. 3–6.

3 $${\text{Magnetic}}\;{\text{model:}}\quad \gamma ( {\text{h)}} = C_{0} + C\left( {1 - \frac{{a^{3} }}{{\left( {a^{2} + h^{2} } \right)^{3/2} }}} \right)$$

4 $${\text{Gaussian}}\;{\text{model:}}\quad \gamma (h) = C_{0} + C\left( {1 - \exp \left( { - 3\left( {\frac{h}{a}} \right)^{2} } \right)} \right)$$

5 $${\text{Spherical}}\;{\text{model:}}\quad \gamma (h) = \left\{ {\begin{array}{*{20}c} {\begin{array}{*{20}l} {{\text{C}}_{0} \;if\;h = 0} \hfill \\ {{\text{C}}_{0} + C\left( {1.5\frac{h}{a} - 0.5\left( {\frac{h}{a}} \right)} \right)^{3} } \hfill \\ {C \; if\;h \ge a} \hfill \\ \end{array} } &\quad {for\;0 < h < a} \\ \end{array} } \right.$$

6 $${\text{Exponential}}\;{\text{model:}}\quad \gamma (h) = C_{0} + C\left( {1 - \exp \left( { - 3\left( {\frac{h}{a}} \right)} \right)} \right)$$

### Cross-validation

The semivariogram model is chosen from a set of mathematical functions that describe spatial relationships. The appropriate model is selected by matching the shape of the curve of the experimental semivariogram to the shape of the curve of the mathematical function. This is clearly illustrated in the “Golden Surfer” software we used in this study. In fact, semivariogram is used in the interpolative kriging technique at its second step. This step is preceded by an exploratory data analysis and followed by a prediction (Gorai and Kumar 2013).

During the exploratory analysis, data consistency was checked, outliers removed and statistical distribution identified. Normal data distribution is decided when the mean and the median are very similar. However, high skewness values indicate the existence of outliers, which are very high or low measured values comparing to the dataset. The outliers are caused by a bad measurement or a bad recording, and must be transformed when they exist.

During the prediction phase, four semivariogram models were plotted in order to select the best-fitted one. Predictive performances of the fitted models are checked on the basis of cross-validationtests. The values of mean error (ME), mean square error (MSE), root mean square error (RMSE), average standard error (ASE) and root mean square standardized error (RMSSE) are estimated to ascertain the performance of the developed models. If the predictions are unbiased, the ME should be almost nil. But because of its weaknesses due to its dependence upon the scale of the data and to its indifference to the wrongness of semivariogram, ME is generally standardized by the MSE, being ideally zero.

However, RMSE and ASE should be calculated to indicate if the prediction errors were correctly assessed in the case where they are close. Otherwise, if the RMSE is less than the ASE (or RMSSE less than 1), then the variability of the predictions is overestimated; and if the RMSE is greater than the ASE (or RMSSE greater than 1), then the variability of the predictions is underestimated. Once the best model is selected, it is used to draw the thematic map that provides the spatial distribution of the parameter to be estimated. All these errors are expressed by Eqs. (7)–(11) below (Goovaerts 1997; Gorai and Kumar 2013).

7 $$ME = \frac{1}{N}\sum\limits_{i = 1}^{N} {\left[ {\mathop Z\nolimits^{*} \left( {\mathop x\nolimits_{i} } \right) - \mathop Z\nolimits^{{}} \left( {\mathop x\nolimits_{i} } \right)} \right]}$$

8 $$MSE = \frac{1}{N}\sum\limits_{i = 1}^{N} {\left[ {\frac{{Z^{*} (x_{i} ) - Z(x_{i} )}}{{\sigma^{2} (x_{i} )}}} \right]}$$

9 $$RMSE = \sqrt {\frac{1}{N}\sum\limits_{i = 1}^{N} {\left[ {\mathop Z\nolimits^{*} \left( {\mathop x\nolimits_{i} } \right) - \mathop Z\nolimits^{{}} \left( {\mathop x\nolimits_{i} } \right)} \right]}^{2} }$$

10 $$ASE = \sqrt {\frac{1}{N}\sum\limits_{i = 1}^{N} {\sigma^{2} (x_{i} )} }$$

11 $$RMSSE = \sqrt {\frac{1}{N}\sum\limits_{i = 1}^{N} {\left[ {\frac{{Z^{*} (x_{i} ) - Z(x_{i} )}}{{\sigma^{2} (x_{i} )}}} \right]}^{2} }$$

where *σ*^*2*^*(x*_*i*_*)* is the Kriging variance for location *x*_*i*_, Z^*^(x_*i*_*)* and *Z(x*_*i*_*)* are the estimated and the measured values of the parameter at the location *x*_*i*_ respectively.

## Abbreviations

ASE: average standard error
ME: mean error
MSE: mean square error
RMSE: root mean square error
RMSSE: root mean square standardized error
