# Erratum to: Lessening the adverse effect of the semivariogram model selection on an interpolative survey using kriging technique

**DOI:** 10.1186/s40064-016-2453-5

**Published:** 2016-07-04

**Authors:** Zakari Arétouyap, Philippe Njandjock Nouck, Robert Nouayou, Franck Eithel Ghomsi Kemgang, Axel Dorian Piépi Toko, Jamal Asfahani

**Affiliations:** Postgraduate School of Science, Technology and Geosciences, University of Yaounde I, P.O. Box 812, Yaounde, Cameroon; Applied Geophysics Division, Head Atomic Energy Commission, P.O. Box 6091, Damascus, Syria

## Erratum to: SpringerPlus (2016) 5:549 DOI 10.1186/s40064-016-2142-4

In the publication of this article (Arétouyap et al. 2016), there was an error in the Competing Interests section. The error: ‘Prof. Asfahani is the Head of Atomic Energy Commission of Syria’ should instead read: ‘Prof. Asfahani is the Head of the Applied Geophysics Division at the Atomic Energy Commission of Syria’.
